# Self-reported anxiety, depression, and health-related quality of life during two years following hospitalization for covid-19: a longitudinal study

**DOI:** 10.1038/s41598-026-48440-3

**Published:** 2026-04-17

**Authors:** Alexandra C. Larsson, Roda Alhasan, Annie Palstam, Lena Rafsten, Hanna C. Persson

**Affiliations:** 1 Department of Clinical Neuroscience and Rehabilitation Medicine, Institute of Neuroscience and Physiology, Sahlgrenska Academy, University of Gothenburg, Gothenburg, Sweden; 2 Department of Occupational Therapy and Physiotherapy, Sahlgrenska University Hospital, Gothenburg, Sweden; 3 School of Health and Welfare, Dalarna University, Falun, Sweden

**Keywords:** COVID-19, HRQoL, Rehabilitation, Recovery of function, Hospitals, Anxiety, Depression, Post-acute COVID-19 Syndrome, Diseases, Health care, Medical research, Psychology, Psychology, Signs and symptoms

## Abstract

Coronavirus disease 2019 (COVID-19) is a respiratory illness caused by severe acute respiratory syndrome coronavirus 2 (SARS-CoV-2). Health-related quality of life (HRQoL) has been observed to decline after COVID-19 hospitalization, with anxiety and depression contributing to reduced HRQoL and emphasizing the need for research on long-term outcomes. The aim of the study was to longitudinally explore self-reported symptoms of anxiety, depression, and associated factors of HRQoL during 2 years after hospital-treated COVID-19, and to compare differences based on age, sex, and initial disease severity. Participants were included from the Life in the Time of COVID study in Gothenburg, comprising individuals hospitalized with COVID-19 during the first and second waves of the pandemic in Sweden. This prospective study includes follow-ups at 3 months, 1 year, and 2 years after discharge, using patient-reported outcomes assessing symptoms of anxiety, depression, HRQoL, and clinical data. The study comprised 125 participants, of whom 111 were eligible for longitudinal analysis. Two years after COVID-19, individuals with initial moderate infection reported higher levels of anxiety (*p* = 0.031) and pain/discomfort (*p* = 0.039) than did those with severe infection. Symptoms of anxiety did not change significantly over the study period. Depressive symptoms increased significantly between 3 and 12 months after COVID-19 (Z = − 2.957, *p* = 0.003). Older age was significantly associated with lower HRQoL (B = − 0.003, 95% CI [–0.004, − 0.001], *p* = 0.010). Symptoms of anxiety remained generally within the normal range throughout the two-year follow-up after hospital-treated COVID-19. Symptoms of depression were lower at 3 months after hospitalization but subsequently stabilized at a higher, yet still normal, level. At two years after COVID-19, lower HRQoL was associated with older age, highlighting the importance of addressing age-related vulnerabilities in long-term follow-up care.

## Introduction

 Coronavirus disease 2019 (COVID-19) is a communicable respiratory disease caused by severe acute respiratory syndrome coronavirus 2 (SARS-CoV-2)^1^. Vaccines and improved treatments have reduced the burden of acute COVID-19^1^. However, post-COVID condition is estimated to affect millions worldwide^2^. According to the WHO’s definition, post-COVID condition is characterized by new or persistent symptoms that manifested 3 months after the initial infection, persist for at least 2 months, and cannot be explained by other causes^3^. Commonly reported symptoms include fatigue, breathlessness, and cognitive impairment, all of which reduce quality of life^1,3^. Individuals who have been hospitalized for COVID-19 have been shown to be more prone to long-term effects of the disease and tend to recover more slowly^4–6^. With this, a need for rehabilitation, defined by the WHO as interventions that optimize functioning and reduce disability in individuals with health conditions, in interaction with their environment^7^, follows. Hospitalized patients often require rehabilitation to varying degrees, as was argued in the context of the COVID-19 pandemic^8^. In line with the proposed continuous needs for rehabilitation, a decline in health-related quality of life (HRQoL) after COVID-19 has recently been shown in a meta-analysis^9^.

Anxiety and depression are prevalent in the general population and are still associated with stigmatization; consequently, some affected individuals may not seek help and may instead characterize their symptoms as manifestations of physical illness^10–12^. Previous studies have reported anxiety and depression as persistent symptoms following COVID-19^13–17^. A higher prevalence of anxiety has been observed among females, younger individuals, and those not hospitalized during the acute phase^15^. An increased burden of depression and anxiety over time after COVID-19 hospitalization has also been documented^14^. In previously hospitalized individuals (the same cohort as the present study), approximately 1 out of 4 reported symptoms of anxiety and/or depression one year after hospital treated COVID-19^17^. Moreover, post-COVID depression and anxiety have been linked to a decline in health-related quality of life (HRQoL)^16^. People with post-COVID syndrome, in combination with symptoms of anxiety or depression and sleep disorders as well as female sex reported lower HRQoL at one year^18^ however, the long-term impact is not fully understood. Understanding factors associated with long-term symptoms of anxiety and depression after COVID-19 may help to improve long-term quality of life in individuals still suffering - years after COVID-19 hospitalization.

The aim of the study was to longitudinally explore self-reported symptoms of anxiety, depression, and associated factors of HRQoL during 2 years after hospital-treated COVID-19, and to compare differences based on age, sex, and initial disease severity.

## Methods

### Study design and participants

This prospective longitudinal study included individuals hospitalized for COVID-19, with the first assessment conducted at hospital discharge, with follow-ups at 3 months, 1 year, and 2 years. Participants comprised the longitudinal Life in the Time of COVID Study in Gothenburg (GOT-LOCO) cohort^19,20^, for which participants were enrolled consecutively from five hospitals in the Västra Götaland region of Sweden between July 2020 and February 2021.

Inclusion criteria were age ≥ 18 years, hospitalization for COVID-19, noncontagious when included, at least 5 days of hospital care for COVID-19, and living independently in the community prior to admission. Exclusion criteria included inability to provide informed consent, severe comorbidities with an anticipated life expectancy of less than 1 year and not being a Swedish resident.

During the hospital stay, all participants received both written and oral information about the study, prior written informed consent was obtained. The study followed the ethical principles of the Declaration of Helsinki and was approved by the Swedish Ethical Review Authority (ref. no. 2020-03046, 2020-03922, 2021-00444 and 2021-03556). The manuscript was prepared in accordance with the STROBE guidelines^21^.

### Data collection

At hospital discharge, clinical characteristics from the hospital stay were extracted from medical records and included length of stay, ICU admission, comorbidities, and COVID-19 severity (dichotomized as moderate or severe) using the WHO Clinical Progression Scale^22^. Moderate infection corresponds to scores of 4–5, and severe infection to scores of 6–9^22^. Comorbidities were categorized according to the ICD-10 version of the Charlson Comorbidity Index (CCI)^23,24^, and classified as none, mild (one comorbidity), or severe (two or more comorbidities).

Following hospital discharge, data was collected at 3 months, 1 year, and 2 years. At the 3-month follow-up, participants completed self-reported patient-reported outcome measures (PROMs) during a scheduled telephone follow-up conducted by a research nurse, trained for the study. At 1 and 2 years after hospital discharge, PROMs were administered to participants by mail or digitally, if preferred, with at least one reminder sent via text message if no response was received. At 2 years additional questions were included regarding patient characteristics and COVID-19–related information, a survey available at the Swedish Healthcare guide online (1177.se) produced by a national working group consisting of medical experts, the national Board of Health and Welfare, and a patient organization. Participants rated the level of difficulty experienced in various domains: activity (not managing daily activities as before COVID-19, such as showering, grocery shopping, and recreational activities), mobility (not managing mobility as before COVID-19, such as walking, moving outdoors), pain and sensitivity, bowel function, smell and taste, swallowing, vision, cardiac function, skin problems, and recurrence of fever. Responses were recorded on a Likert scale from 0 (no difficulties) to 4 (severe difficulties). The questionnaire has previously been used in a national cohort to evaluate consequences of COVID-19^25^.

Self-reported symptoms of anxiety and depression were reported using the Hospital Anxiety and Depression Scale (HADS). Anxiety and depression scores both range from 0 to 21, with higher scores indicating greater distress. Scores ≤ 7 are considered normal, 8–10 elevated, and ≥ 11 concerning, and in the present study a subscore of ≥ 8 was used as a proxy for anxiety and / or depression^12,26^. HADS is considered a valid instrument and has demonstrated good internal consistency, with a mean Cronbach’s alpha of 0.83 for the anxiety scale and 0.82 for the depression scale^26^.

The EuroQol-5 Dimension (EQ-5D) was used to self-report HRQoL across five dimensions: mobility, self-care, usual activities, pain/discomfort, and anxiety/depression, each rated on three levels (1 = no problems, 2 = some problems, 3 = extreme problems). A response level of ≥ 2 on any dimension was used as a proxy for reduced HRQoL. The EQ Visual Analogue Scale (VAS) measures overall health from 0 (“worst imaginable health state”) to 100 (“best imaginable health state”). Responses are combined into an EQ-5D index ranging from − 0.594 to 1.0, with higher numbers indicating better HRQoL^27^. Both the EQ-5D index and EQ-VAS have demonstrated good validity and sufficient reliability for group-level comparisons in clinical populations^28^.

### Statistical analysis

Demographic data are presented as percentages, mean ± standard deviation (SD), or median and interquartile range (IQR), as appropriate. The level of statistical significance was set at *p* ≤ 0.05. Group comparisons were conducted based on sex, age group (≥ 65 or < 65 years), and COVID-19 severity (moderate or severe). Dropout analyses were performed using the chi-square test and the Mann–Whitney U-test.

The Friedman test was used to evaluate changes across the three follow-up time points, and the Wilcoxon signed-rank test was applied for pairwise comparisons between time points. For longitudinal analyses, only individuals who participated in the assessments at 3 months, 1-year and 2-year follow-ups were eligible to be included (*n* = 111). Due to internal dropouts, the number of participants in each analysis varies. The Z-value in the shift analysis represents standardized differences between the distributions of the groups.

Multivariable linear regression analysis was used to analyze predictors at hospital discharge potentially associated with HRQoL 2 years after COVID-19. The EQ-5D index at 2 years after hospital discharge was used as the dependent variable. Independent variables assessed at hospital discharge included age, sex, COVID-19 severity (moderate/severe), ICU treatment (yes/no), total length of hospital stay (days), and comorbidities (none/mild/severe). Independent variables were tested for multicollinearity using Spearman correlation (Rho > 0.7). Initial analysis for assumptions for linear regression—normality, linearity, absence of multicollinearity, and lack of influential cases—were performed. Initial analyses included scatterplots for continuous data and boxplots for categorical data.

All analyses were conducted using SPSS Statistics version 28 (IBM Corporation, Armonk, NY, USA). Microsoft Excel and SankeyMATIC.com were used for visual presentations.

## Results

**Fig. 1:**
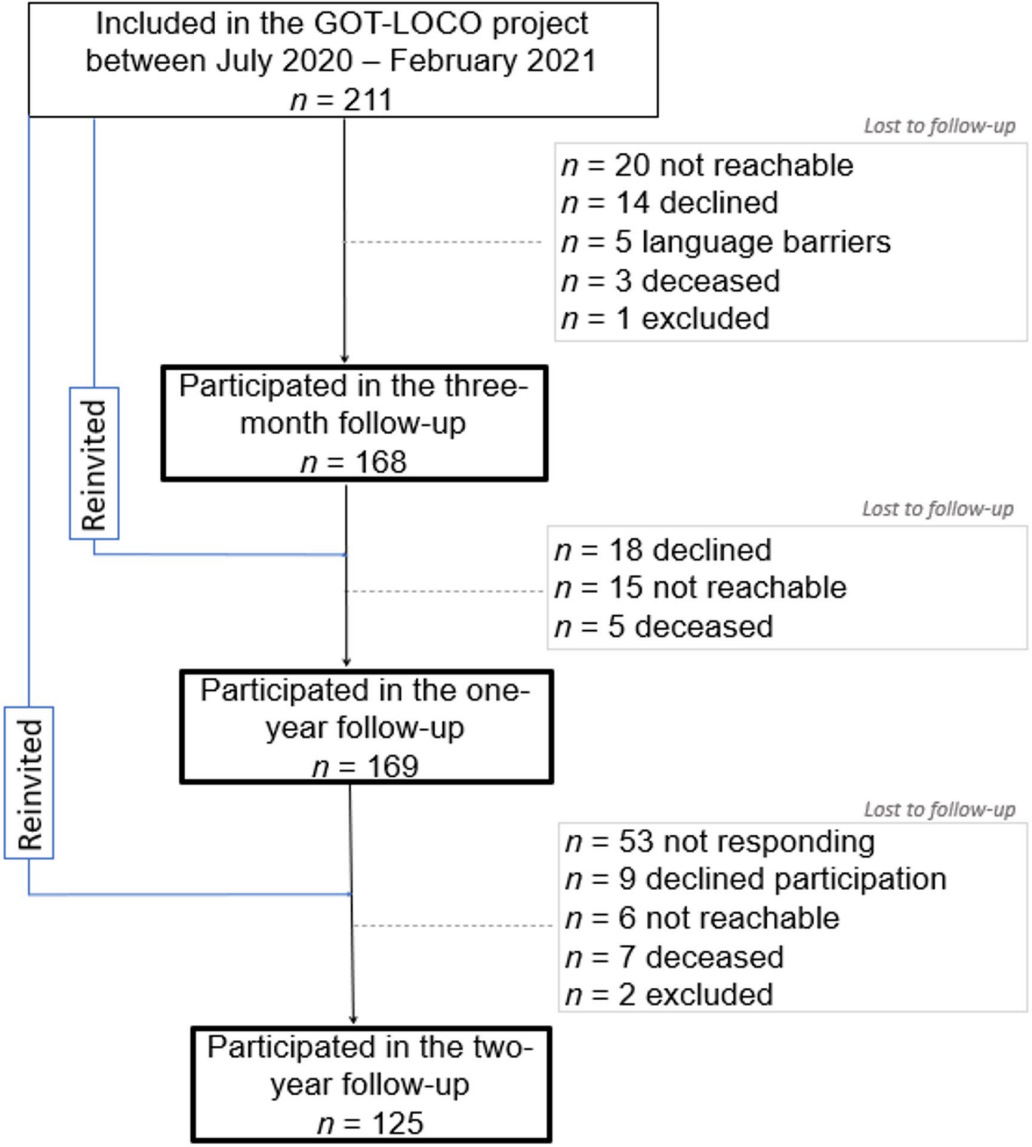
Flow-chart of participants.

A total of 211 participants were enrolled in the GOT-LOCO project at the acute hospital care. Of these, 168 participated in the 3-month follow-up, 169 in the 1-year follow-up, and 125 in the 2-year follow-up (Fig. 1). Of the 211 participants, a total of 111 participants completed all follow-ups at 3 months, 1 year and 2 years. No significant differences were found between participants (*n* = 111) and nonparticipants (*n* = 83) in terms of sex, COVID-19 severity, level of hospital care (ICU yes/no), or length of hospital stay. However, the mean age was higher in participants (66 ± 11) than in nonparticipants (62 ± 15; *p* = 0.002).

Among the 125 participants in the 2-year follow-up, 68% (*n* = 85) were men. The vast majority, 96% (*n* = 118), reported having received at least one or more doses of COVID-19 vaccine since their hospitalization. Two-thirds of the participants (62%, *n* = 77) lived in a shared household, and over one-third (*n* = 45) required assistance with daily activities. Additionally, 17% (*n* = 21) reported ongoing rehabilitation for COVID-19–related impairments 2 years after discharge (Table 1).

**Table 1:** Characteristics of participants at the 2-year follow up. *n* = 125.

At hospital discharge	Age, years, mean ± SD	66 ± 12
	Sex, male, number (%)	85 (68)
	LOS, days, mean ± SD	32 ± 32
	Treated at ICU, number (%)	64 (51)
	LOS in ICU, number (%)	19 ± 19
	COVID severity, number (%)	
	Moderate	37 (30)
	Severe	88 (70)
	CCI, number (%), *n* = 124	
	None	45 (36)
	Mild	59 (48)
	Severe	20 (16)
At the 2-year follow-up	Dependent on ADL assistance, number (%)	45 (36)
	Living situation, number (%)	
	Alone	48 (38)
	Shares household with another adult	62 (50)
	Shares household with children	12 (10)
	Shares household with both children and another adult	3 (2)
	Vaccination, number (%), *n* = 123	
	No	5 (4)
	Yes (number of doses unknown)	2 (2)
	Yes, two doses	12 (10)
	Yes, multiple doses	104 (84)

*ADL* activities of daily living, *CCI* Charlson comorbidity index: none = 0, mild = 1–2, severe = ≥ 3, *ICU* intensive care unit, *LOS* length of hospital stay, SD standard deviation, COVID severity is reported according to the WHO Clinical Progression Scale^29^.

**Fig. 2:**
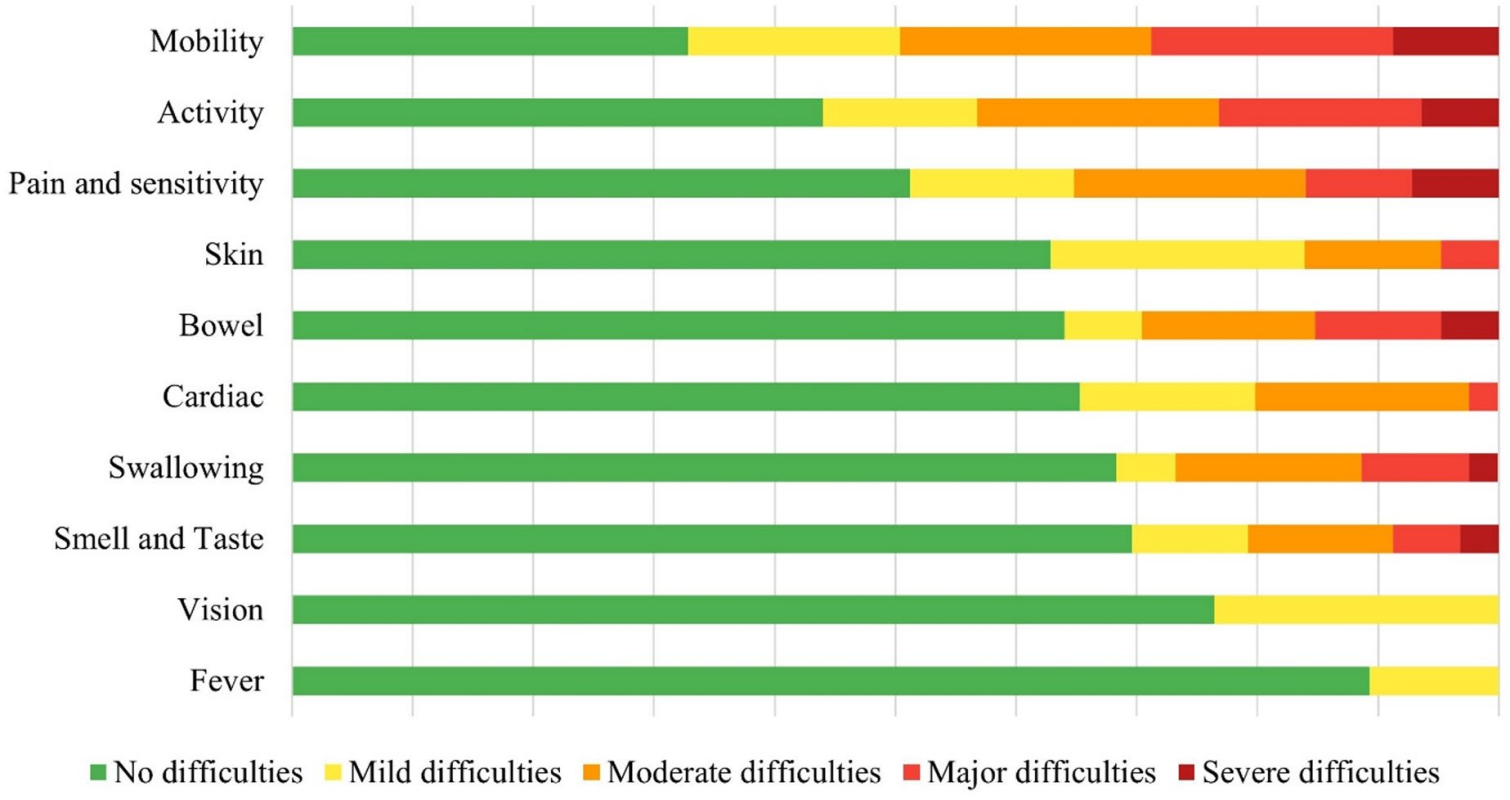
Self-reported areas of difficulty among participants (*n* = 108) 2 years after hospitalization for COVID-19. With answers rated from no difficulties to severe difficulties in each domain. Presented using 100% stacked bars in a descending order.

At the 2-year follow-up, the most common self-reported difficulties were related to the domains: mobility, activity, pain and sensitivity. No or mild difficulties were reported regarding fever and vision problems (Fig. 2). A small number of participants (*n* = 8) reported a high symptom burden (> 25), while there were no participants who reported not having symptoms at all.

30% of participants (*n* = 37) reported symptoms of anxiety, and 24% (*n* = 29) reported symptoms of depression at the 2-year follow-up. No significant differences in self-reported anxiety or depression were found based on sex or age (Table 2). Participants with moderate initial COVID-19 severity reported levels of anxiety higher than those reported by individuals hospitalized with severe COVID-19 (*p* = 0.031). Regarding HRQoL, participants with moderate initial COVID-19 also reported higher levels of pain and discomfort than those reported by individuals hospitalized with severe COVID-19 (*p* = 0.039). A significant difference was also observed between participants over and under 65 years of age regarding mobility difficulties, where older individuals reported mobility difficulties to a higher extent (*p* = 0.001).

**Table 2:** Outcomes of self-reported anxiety, depression, and health-related quality of life at 2 years after COVID-19, depending on sex, age, and COVID severity.

Outcome	Overall *n* = 125	Sex		*P*	Age (years)		*P*	Severity		*P*
		Male *n* = 85	Female *n* = 40		≥ 65 *n* = 53	< 65 *n* = 72		Moderate *n* = 37	Severe *n* = 88	
HADS-A score *n* = 122										
Median (IQR)	4 (1–8)	4 (1–8)	3 (2–8)	0.638	4 (1–8)	3 (1–9)	0.521	6 (8)	3 (6)	**0.031**
HADS-D score *n* = 122										
Median (IQR)	3 (1–7)	3 (1–8)	2.5 (1–5)	0.487	3 (1–7)	2 (1-7.5)	0.956	4 (2–8)	2 (1–7)	0.095
Equation 5D										
Mobility *n* = 123										
Median (IQR)	1 (1–2)	1 (1–2)	2 (1–2)	0.158	2 (1–2)	1 (1–2)	**< 0.001**	2 (1–2)	1 (1–2)	0.214
Self-care *n* = 123										
Median (IQR)	1 (1–1)	1 (1–1)	1 (1–1)	0.894	1 (1–1)	1 (1–1)	0.173	1 (1–1)	1 (1–1)	0.468
Activity *n* = 122										
Median (IQR)	1 (1–2)	1 (1–2)	1 (1–2)	0.287	1 (1–2)	1 (1–2)	0.692	1 (1–2)	1 (1–2)	0.629
Pain / discomfort *n* = 121										
Median (IQR)	2 (1)	2 (1–2)	2 (1–2)	0.486	2 (1–2)	2 (1–2)	0.076	2 (2–2)	2 (1–2)	**0.039**
Anxiety / depression *n* = 123										
Median (IQR)	1 (1–2)	1 (1–2)	2 (1–2)	0.105	1 (1–2)	1 (1–2)	0.384	2 (1–2)	1 (1–2)	0.161
EQ-VAS (%)										
Median (IQR) *n* = 105	62 (45–80)	62,5 (45–80)	61 (40–89)	0.863	61 (45–80)	65 (47–80)	0.784	60 (50–80)	63.5 (40–80)	0.704

Significant *P*-values are presented in bold. *A* anxiety, *D* depression, *Eq. 5D* EuroQol-5 Dimension, *EQ-VAS* EuroQol Visual Analogue Scale, *HADS* Hospital anxiety and depression scale.

Health-related quality of life declined slightly between the 1- (median 0.88) and 2-year (median 0.86) follow-ups- with lower EQ-5D index (*p* = 0.046) with increased proportions of participants reporting moderate problems in mobility, self-care, and usual activities (Table 3).

**Table 3:** Health-related quality of life at 1 and 2 years after COVID-19.

	1-year follow-up	2-year follow-up
	*n* = 117	*n* = 123
Eq. 5D		
Equation 5D Index, Median (IQR)	0.88 (0.79–93)	0.86 (0.75–0.93)

	*n* = 114	*n* = 120
Mobility, %		
Moderate problems	43.1	49.6
Extreme problems	–	–
Self-care, %		
Moderate problems	6.9	13.8
Extreme problems	–	0.8
Usual Activity, %		
Moderate problems	20.7	34.4
Extreme problems	2.6	1.6
Pain/Discomfort, %		
Moderate problems	56.1	53.7
Extreme problems	9.6	12.4
Anxiety/Depression, %		
Moderate problems	35.3	39.0
Extreme problems	3.4	4.9

	*n* = 108	*n* = 105
VAS		
Median (IQR)	70 (53–85)	62 (45–80)
Mean (SD)	68 ± 20	64 ± 20

Data are presented as count and percentages (*n*, %).

*EQ-5D* EuroQol-5 Dimension, *EQ-VAS* EuroQol-Visual Analog Scale.

**Fig. 3:**
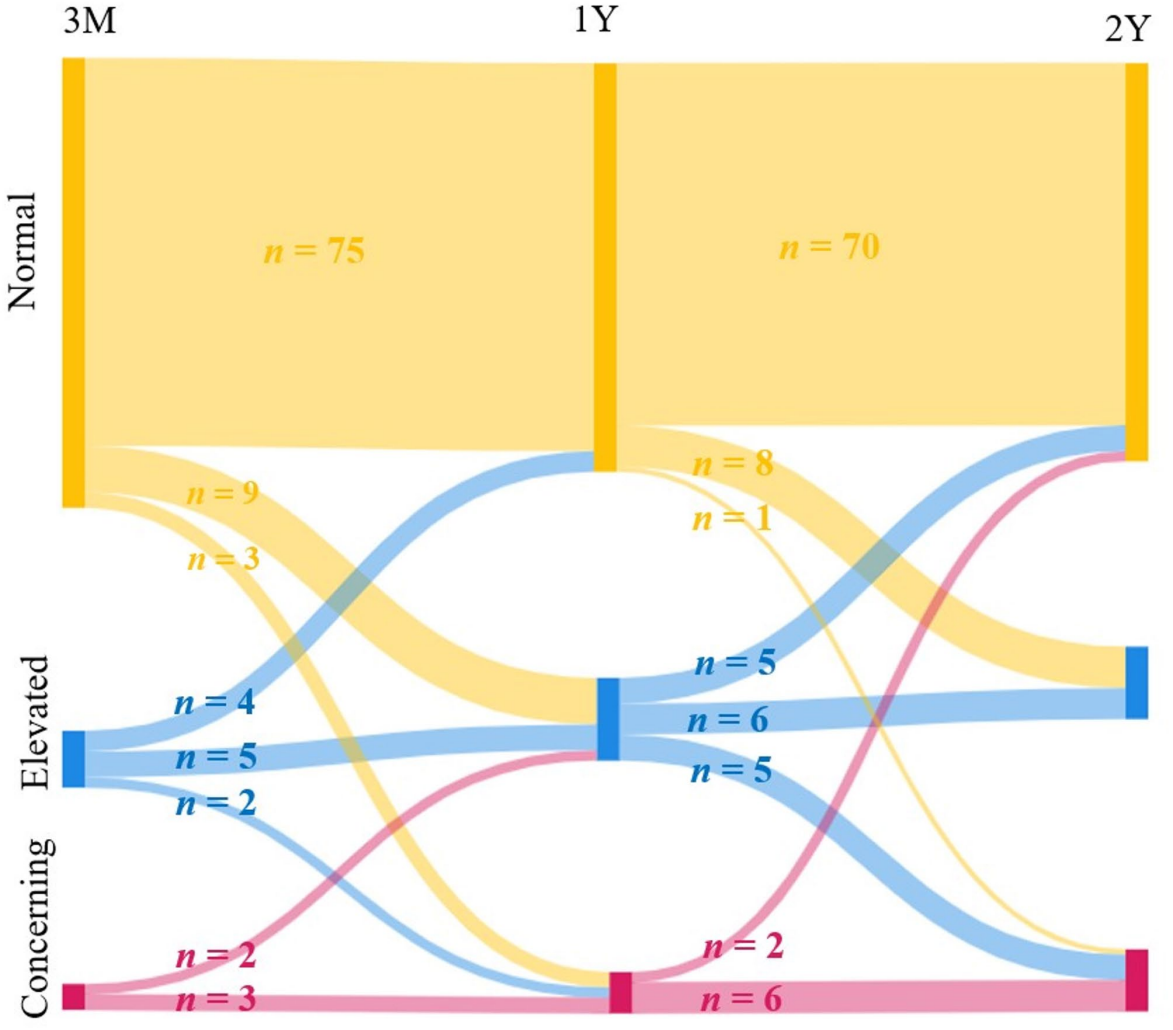
The change in **s**elf-reported levels of depression (*n* = 103) at the 3-month, 1-year, and 2-year follow-ups, with the number of participants in each category at each time point.

Self-reported symptoms of depression were low 3 months after hospital discharge (median 2, IQR 1–5) but increased for some participants throughout the follow-up period (1 year: median.3 IQR 1–7, year 2: median 3 IQR 1–7.5) Longitudinal analyses showed significant change in symptoms of depression during the study period (*p* = 0.014). Pairwise comparisons showed a significant increase in depressive symptoms from 3 months to 1 year (Z = − 2.913, *p* = 0.004) and from 3 months to 2 years (Z = − 2.726, *p* = 0.006), indicating a worsening trend during the first-year post discharge. However, no significant difference was found between 1 and 2 years (Z = − 0.677, *p* = 0.498) (Fig. 3).

**Fig. 4:**
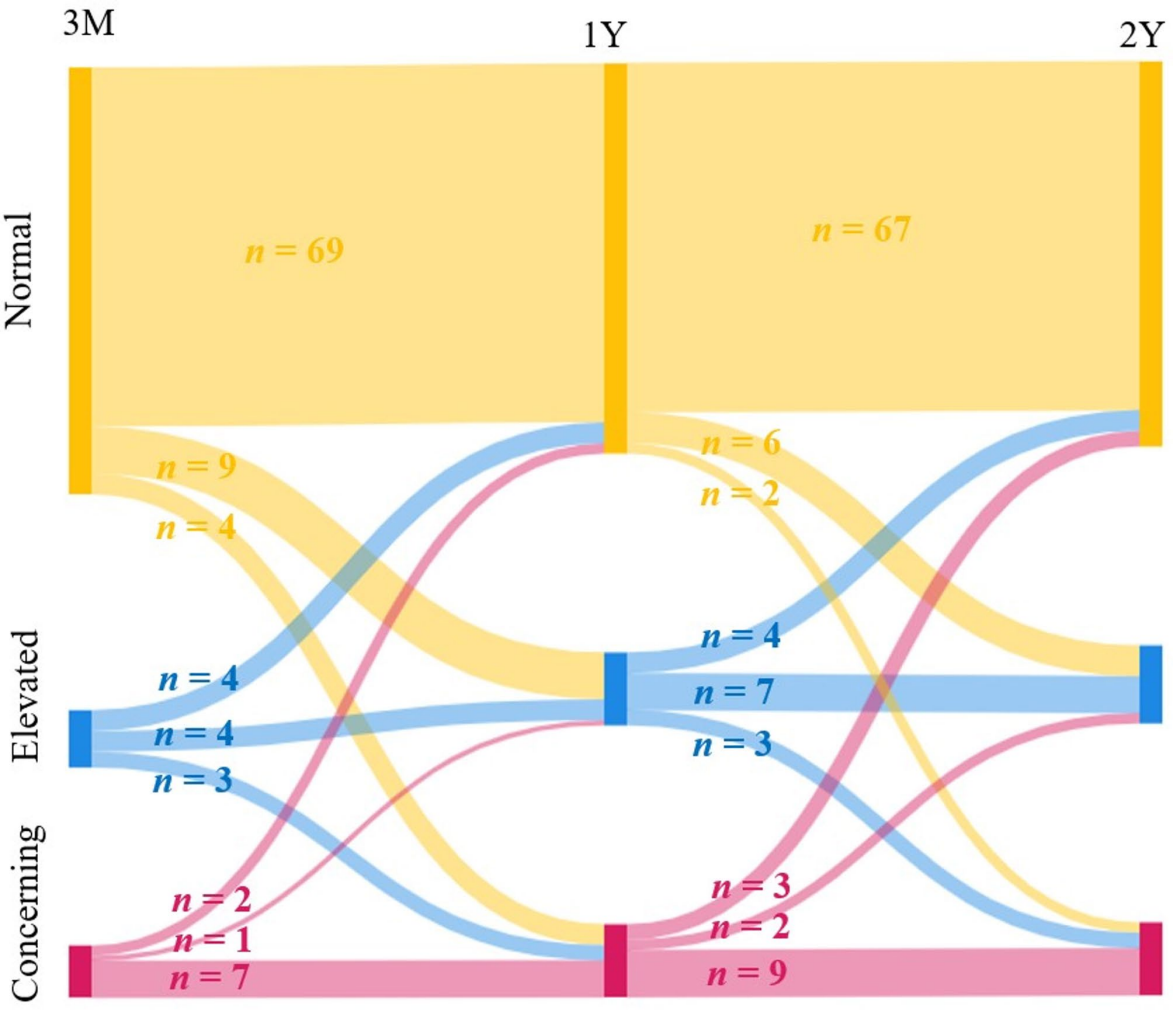
The change in self-reported levels of anxiety (*n* = 103) at the 3-month, 1-year, and 2-year follow-ups, with the number of participants in each category at each time point.

Self-reported anxiety levels remained largely stable over the follow-up period: 3 months (median: 4, IQR: 1–7), 1 year (median: 4, IQR: 1–8), and 2 years (median: 4, IQR: 2–8) after hospital discharge. No statistically significant difference was observed across the time points (*p* = 0.535). These results are visualized in Fig. 4.

Regarding HRQoL at 2 years after hospital treated COVID-19, older age was significantly associated with lower EQ-5D index (B = − 0.003, 95% CI [–0.004, − 0.001], *p* = 0.010), indicating that older participants reported lower HRQoL. Sex, COVID-19 severity, ICU admission, comorbidities, and hospital length of stay did not contribute significantly to the model (Table 4).

**Table 4:** Explanatory factors at hospital discharge associated with health-related quality of life 2 years after COVID-19 infection (*n* = 116).

Variables	Reference	Unstandardized coefficients		*p*-value	95% CI for (B)	
		B	Std. Error		Lower	Upper
Sex	Females (0)	−0.037	0.025	0.140	−0.087	0.012
Age, years		−0.003	0.001	**0.010**	−0.004	−0.001
Covid severity	Moderate (0)	0.007	0.033	0.832	−0.059	0.073
ICU	No ICU (0)	−0.014	0.032	0.668	−0.077	0.050
Comorbidities		−0.005	0.009	0.593	−0.022	0.013
Length of hospital stay		0.000	0.000	0.239	−0.001	0.000

Reference categories are shown for categorical variables. Continuous variables (age, comorbidities, and hospital stay) were entered as linear terms. *B* unstandardized coefficient.

## Discussion

In this longitudinal study including a 2-year follow-up after hospital treated COVID-19 infection, self-reported symptoms of anxiety remained stable whereas self-reported symptoms of depression initially were low, increased during the first year but were stable (within normal levels) thereafter. Older age was significantly associated with lower HRQoL 2 years after COVID-19. Individuals with moderate disease severity reported higher levels of anxiety and pain/discomfort than did those with severe disease.

This finding contrasts with previous research that reported individuals with severe COVID-19 had more anxiety than less severe cases^30^. Why individuals with moderate COVID-19 report higher levels of anxiety, pain, and discomfort remains unclear. One possible explanation is post-traumatic growth (PTG) among individuals who experienced severe COVID-19. PTG refers to positive psychological change, such as increased personal strength and altered life priorities^31^, described in COVID-19 survivors within the first year^30,32,33^. Another possible explanation could be that individuals with severe COVID-19 may have received more rehabilitation and support, including tools and strategies to manage their symptoms, from healthcare services. Previous research has also highlighted the crucial role of hospital care for mental and emotional outcomes, emphasizing the importance of feeling supported by the healthcare system as a protective factor for mental health^34^.

In the present study, 30% of participants continued to experience symptoms of anxiety 2 years after COVID-19. No significant differences in anxiety were found based on sex or age, which contrasts with previous research showing higher anxiety levels among women^34,35^. In the present cohort, men reported higher median symptoms of anxiety than the general population, while women’s scores were similar to their population norms^35^. However, it is important to note that anxiety levels prior to infection were unknown. This reversal of the commonly observed sex pattern warrants further research and clinical attention.

Like the scores for anxiety, the 2-year median scores concerning self-reported depression resembled those reported in the general Swedish population, in both men and women^35^. This suggests that depressive symptoms are lower during the early recovery phase, perhaps as a result of the post-traumatic life event of being hospitalized for COVID-19 and eventually return to levels similar to the population norm.

HRQoL 2 years after COVID-19 revealed that older individuals more frequently reported mobility problems, consistent with age-related expectations^36^. In comparison with the median values of the general Swedish population, higher levels of mobility problems were reported by the COVID-19 cohort. For example, 50% of participants in this cohort reported moderate mobility problems compared with 10% in a general population^37^. Notably, participants who had experienced moderate initial COVID-19 severity reported significantly more pain and discomfort than those with severe illness (*p* = 0.039). Although this study did not examine potential associations between moderate COVID-19 severity, elevated anxiety, and increased pain and discomfort, such connections may be important. Around half of the participants reported moderate pain and discomfort, and one in ten reported extreme problems — both slightly higher than in the general population^37^. These findings were further supported by the participants’ responses to the general questions. The domains with the greatest difficulties were mobility, activity, pain and sensitivity, showing similar patterns, although the questions were not fully aligned with the EQ-5D domains. That pain/discomfort are commonly affected domains after COVID-19 has recently been confirmed by a review article^9^. The high prevalence of pain and discomfort among individuals previously hospitalized with COVID-19 may be relevant for a wide range of healthcare professionals, including those who regularly encounter this patient group or work with pain management and rehabilitation.

The regression model identified older age as significantly associated with lower HRQoL 2 years after hospitalization for COVID-19, independent of sex, initial disease severity, ICU admission, comorbidities, or length of hospital stay. This suggests that COVID-19 may have lasting impacts on older individuals regardless of pre-existing health status. The natural decline in fitness with ageing should be considered^36^ but does not lessen the impact that COVID-19 has on the older population^38^. Together, these findings highlight the importance of long-term follow-up and targeted rehabilitation interventions to support older adults in their recovery after severe COVID-19.

The strengths of this study include its longitudinal design, which followed hospitalized participants from discharge and over a 2-year period. The outcomes were reported using well-established PROMs. This approach allowed observation of changes in symptoms, anxiety, depression, and self-reported HRQoL. This study had some limitations. As this study used a consecutive inclusion process, some caution is warranted when generalizing the findings: certain individuals hospitalized for COVID-19 were overlooked during inclusion, mainly because of heavy workloads in the clinics. The risk of reporting bias as well as response shift^39^ also needs to be taken into consideration, as this study relies on PROMs. Additionally, the absence of a control group complicates charting the occurrence and burden of symptoms in comparison with other individuals.

## Conclusion

Symptoms of anxiety remained generally within the normal range throughout the two-year follow-up after hospital-treated COVID-19. Symptoms of depression were lower at 3 months after hospitalization but subsequently stabilized at a higher, yet still normal, level. At 2 years after COVID-19, lower HRQoL was associated with older age, highlighting the importance of addressing age-related vulnerabilities in long-term follow-up care and rehabilitation.

## Data Availability

The datasets generated during and/or analyzed during the current study are not publicly available due to ethical restrictions, according to the Swedish regulation the permission to use data is only for what has been applied for and then approved by the Swedish Ethical Review Authority but are available from the corresponding author on reasonable request.

## Abbreviations

CI: Confidence interval
EQ-5D: EuroQol-5 dimension
HADS: Hospital anxiety and depression scale
HRQoL: Health related quality of life
ICU: Intensive care unit
IQR: Inter quartile range
LOS: Length of hospital stay
SD: Standard deviation
PROMs: Patient reported outcome measures
PTG: Post traumatic growth
VAS: Visual analog scale
